# 酪氨酸激酶抑制剂治疗成人初发慢性髓细胞性白血病患者发生严重血细胞减少的危险因素

**DOI:** 10.3760/cma.j.cn121090-20251023-00478

**Published:** 2026-03

**Authors:** 国蓉 柴, 露 于, 宗儒 李, 亚溱 秦, 红霞 石, 悦云 赖, 悦 侯, 倩 江

**Affiliations:** 北京大学人民医院、北京大学血液病研究所、国家血液系统疾病临床医学研究中心，北京 100044 Peking University People's Hospital, Peking University Institute of Hematology, National Clinical Research Center for Hematologic Disease, Beijing 100044, China

**Keywords:** 白血病，髓样，慢性, 酪氨酸激酶抑制剂, 血细胞减少, Leukemia, myeloid, chronic, Tyrosine kinase inhibitors, Cytopenia

## Abstract

**目的:**

探讨初发慢性髓细胞性白血病慢性期（CML-CP）患者服用酪氨酸激酶抑制剂（TKI）期间发生严重血细胞减少的影响因素。

**方法:**

纳入2006年11月至2025年1月在北京大学人民医院确诊并服用伊马替尼、尼洛替尼或氟马替尼的成人CML-CP连续病例。采用二元Logistic回归模型分析发生严重血细胞减少（按CTCAE5.0分级，关注≥3级白细胞或血小板减少）的影响因素。

**结果:**

共收集1 906例患者数据，中位年龄41（18～83）岁，男性1 141例（59.9％），一线服用伊马替尼1 542例（80.9％），尼洛替尼256例（13.4％），氟马替尼108例（5.7％）。186例（9.8％）患者在TKI中位治疗1（0.2～3）个月发生严重血细胞减少，且以血小板减少为主，其中4级血细胞减少68例（3.6％），持续0.6（0.1～10.6）个月。三种TKI治疗组≥3级血细胞减少的发生率差异无统计学意义，但二代TKI组（包括尼洛替尼和氟马替尼）4级血细胞减少发生率相较伊马替尼组显著升高（7.3％对2.7％，*P*＝0.001）。多因素分析显示，女性、HGB低和WBC高（或脾大）是TKI相关的严重血细胞减少的危险因素。根据危险因素将服用伊马替尼患者分为低危和高危两组，将服用二代TKI患者分为低危、中危和高危三组，各组≥3级和4级血细胞减少发生率差异均有统计学意义（均*P*<0.001）。

**结论:**

严重血细胞减少是TKI治疗的CML-CP患者常见的不良事件，二代TKI 4级血细胞减少发生率更高。女性、HGB低、WBC高和脾大是TKI相关严重血细胞减少的影响因素。针对高危人群在治疗早期应严密监测。

## Abstract (en)

**Objective:**

To explore the variables associated with severe cytopenia in patients with chronic phase chronic myeloid leukemia（CML-CP）receiving initial tyrosine kinase inhibitor（TKI）therapy.

**Methods:**

Data from consecutive patients aged ≥18 years with CML-CP who received imatinib, nilotinib, or flumatinib at Peking University People's Hospital between November 2006 and January 2025 were retrospectively reviewed. Binary logistic regression models were applied to analyze the variables associated with severe cytopenia（according to Common Terminology Criteria for Adverse Events version 5.0, with a focus on grade 3/4 leukocytopenia or thrombocytopenia）.

**Results:**

This study included 1 906 patients initially receiving imatinib（*n*＝1 542, 80.9％）, nilotinib（*n*＝256, 13.4％）, and flumatinib（*n*＝108, 5.7％）. The median age was 41（range, 18–83）years, and 1 141（59.9％）patients were male. At a median of 1（range, 0.2–3）month, 186（9.8％）patients developed grade 3/4 cytopenia, primarily thrombocytopenia, and persisted for 0.6（range, 0.1–10.6）months, including 68（3.6％）patients who developed grade 4 cytopenia. No significant difference in the incidence of grade 3/4 cytopenia was observed among patients receiving the three TKIs, whereas grade 4 cytopenia was more common in those receiving the second-generation（2G）TKI（nilotinib and flumatinib）compared with imatinib（7.3％ *vs* 2.7％, *P*＝0.001）. Multivariate analysis revealed that female sex, decreased hemoglobin level, and increased white blood cell count（or splenomegaly）were significantly associated with the higher incidence of severe cytopenia in patients receiving TKI. Based on the adverse variables, patients receiving imatinib were categorized into low- and high-risk groups, and those receiving 2G-TKI were classified into low-, medium-, and high-risk groups with a significant difference in the incidence of grades 3/4 and 4 cytopenia（*P*<0.001）.

**Conclusion:**

Severe cytopenia is a common adverse event in patients with CML-CP receiving TKI therapy. Grade 4 cytopenia is more common in those receiving 2G-TKI compared with imatinib. High-risk factors for severe cytopenia associated with TKIs included female sex, decreased hemoglobin level, increased white blood cell count, and splenomegaly at initial diagnosis. Close monitoring is necessary during the early phase of TKI-therapy for the high-risk group.

酪氨酸激酶抑制剂（TKI）已经成为慢性髓细胞性白血病慢性期（CML-CP）患者的一线治疗[1]–[2]。国内获批一代TKI伊马替尼、二代TKI尼洛替尼和氟马替尼，尽管耐受性良好，但仍存在不良反应，包括严重的血细胞减少。根据欧洲白血病网（ELN）和美国国立综合癌症网络（NCCN）关于CML治疗管理和不良事件预防的建议推荐，≥3级白细胞、中性粒细胞和血小板减少是CML患者中断治疗的指征[3]–[4]。数项RCT研究报道了伊马替尼与尼洛替尼或氟马替尼比较，≥3级血液学不良反应的发生率相近[5]–[6]，但真实世界数据报道较少。初诊时预测严重血细胞减少发生的可能性，有助于TKI治疗中的优化管理，包括严密监测高危人群，早期识别严重血细胞减少的发生并予以及时干预。为此，我们回顾性分析北京大学人民医院诊治的CML-CP患者，评估严重血细胞减少的发生率和相关因素。

## 病例与方法

一、病例

回顾性收集2006年11月至2025年1月于我院以伊马替尼、尼洛替尼和氟马替尼为一线治疗成人CML-CP病例资料。诊断和分期参照ELN关于CML诊断和分期标准[2],[7]–[9]。排除初诊到治疗超过6个月、不规律随访患者。收集患者信息，包括性别、初诊时年龄、脾脏大小（肋下厘米数）、全血细胞计数、外周血原始细胞及嗜碱性粒细胞比例、合并症、一线TKI种类和剂量、治疗期间严重血液学不良反应的发生情况，本研究仅关注按CTCAE 5.0进行的分级≥3级白细胞和血小板减少。本研究经北京大学人民医院伦理委员会批准（审批号：2022PHB103-001），豁免知情同意。

二、治疗

TKI的选择根据患者的年龄、疾病危险度、合并症、合并用药、经济情况以及治疗目标等，由患者和医师共同决定一线TKI药物。剂量调整参照ELN推荐[2],[7]–[9]。初始剂量为伊马替尼400 mg/d，尼洛替尼300 mg每日两次，或氟马替尼600 mg/d。当发生≥3级的白细胞或血小板减少时停药，直至WBC>2.0×10^9^/L，PLT>50×10^9^/L时恢复用药。若停药时间超过2周，恢复用药时剂量减少1/4～1/3（伊马替尼300 mg/d，尼洛替尼450 mg/d或氟马替尼400 mg/d），之后根据血象和治疗反应调整药量。

三、监测及随访

监测方法和频率参照ELN推荐[2],[7]–[9]。血液学：治疗前3个月每1～2周进行血细胞分类计数；治疗第3～6个月，每1～2个月检测1次；之后每3个月检测1次。细胞遗传学：采用骨髓进行染色体核型分析，每3～6个月评估1次，直至获得完全细胞遗传学反应（CCyR），当患者出现治疗失败或疾病进展时，重启骨髓细胞遗传学检测。分子学反应：采用外周血标本，利用实时定量PCR（qRT-PCR）监测BCR-ABL水平，每3个月监测1次直至达到主要分子学反应（MMR），之后每3～6个月监测1次；当出现警告或治疗失败时，通过Sanger测序检测ABL激酶区突变情况。采用门诊、查阅病历或电话联系方式进行随访，随访截止时间为2025年5月。

四、评估指标

血液学不良反应：根据CTCAE 5.0进行分级，3级白细胞和血小板减少分别定义为WBC<2.0×10^9^/L，PLT<50×10^9^/L。4级白细胞和血小板减少分别定义为WBC<1.0×10^9^/L，PLT<25×10^9^/L。

五、统计学处理

1. 描述性统计：对患者信息进行描述性统计，分类变量用频数和频率进行描述，连续变量用*M*（范围）或*M*［四分位间距（*IQR*）］表示。对数据进行组间比较时，连续变量正态分布数据采用*t*检验或单因素方差分析，非正态分布数据采用Mann-Whitney *U*或Kruskal-Wallis *H*非参数检验，组间比较采用Dunnett's T3检验；分类变量进行卡方检验或Fisher精确概率法，组间比较采用Bonferroni校正显著性阈值。通过倾向性评分匹配（PSM）减少一线TKI药物选择偏倚，使用年龄、性别、全血细胞计数、外周血原始细胞及嗜碱性粒细胞百分比、ELTS评分、合并症等变量为协变量，以1∶2或1∶3比例匹配，卡钳值设置为0.2。*P*<0.05为差异有统计学意义。

2. 分析≥3级血细胞减少的相关因素：采用受试者工作特征曲线（ROC）确定连续变量对于发生严重血细胞减少的最佳截断值，若截断值无法确定，则用中位数或公认值代替。应用单、多因素二元Logistic回归模型分析与≥3级血细胞减少显著相关的变量，单因素分析中*P*<0.2的变量纳入多因素分析。依据多因素分析中显著变量的回归系数赋分建立预测模型。本研究采用SPSS22.0、R4.0.2和Origin 2024软件进行数据分析和绘图。

## 结果

一、患者特征

共纳入1 906例TKI治疗的成人CML-CP患者，中位年龄41（18～83）岁，男性1 141例（59.9％）。ELTS评分低、中和高危分别为1 234例（64.7％）、456例（23.9％）和152例（8％）。627例（32.9％）有合并症。一线服用伊马替尼1 542例（80.9％），二代TKI（包括尼洛替尼和氟马替尼）364例（19.1％），其中尼洛替尼256例（13.4％）、氟马替尼108例（5.7％）。三组人群的年龄、初诊时WBC、HGB、PLT、外周血原始细胞、脾脏肋缘下厘米数、合并症、ELTS危险度和随访时间的差异均有统计学意义（均*P*<0.05）。组间比较结果显示，服用伊马替尼人群的初诊时WBC［*P_1_*（与尼洛替尼组比较）＝0.030；*P_2_*（与氟马替尼组比较）＝0.042］、PLT（*P_1_*＝0.182；*P_2_*＝0.042）、外周血原始细胞（*P_1_*＝0.048；*P_2_*<0.001）最低，HGB最高（*P_1_*＝0.001；*P_2_*＝0.084），脾脏肿大最不显著（*P_1_*<0.001；*P_2_*＝0.913），ELTS评分中、高危患者比例最低（*P_1_*＝0.015；*P_2_*＝0.083），随访时间最长（*P_1_*<0.001；*P_2_*<0.001）；服用尼洛替尼人群初诊时年龄最小（与伊马替尼组比较，*P*<0.001；与氟马替尼组比较，*P*<0.001），有合并症的比例最低（与伊马替尼组比较，*P*＝0.001；与氟马替尼组比较，*P*＝0.008）。三组间性别、初诊时外周血嗜碱性粒细胞比例差异均无统计学意义（表1）。

**表1:** 纳入研究的1 906例初发成人慢性髓细胞性白血病患者临床特征及三组临床特征比较

特征	总体 （1 906例）	伊马替尼组 （1 542例）	尼洛替尼组 （256例）	氟马替尼组 （108例）	统计量 （*χ^2^*/*F*/*H*）	*P*值
年龄［岁，*M*（范围）］	41（18～83）	42（18～83）	34（18～73）	44（18～71）	47.929	<0.001
男性［例（％）］	1 141（59.9）	938（60.8）	147（57.4）	56（51.9）	4.120	0.127
WBC［×10^9^/L，*M*（范围）］	103.6（2.1～723.7）	99.6（2.1～723.7）	142.1（6.2～667.3）	157.1（10.3～659.9）	12.505	0.002
HGB［g/L，*M*（范围）］	116（43～340）	118（43～340）	110（60～166）	113（56～174）	6.615	0.001
PLT［×10^9^/L，*M*（范围）］	410.5（22～3 773）	406（22～3 773）	430（28～2 887）	460（79～1 906）	6.693	0.035
外周血原始细胞［％，*M*（范围）］	1（0～14）	1（0～14）	1（0～13）	2（0～13）	31.633	<0.001
外周血嗜碱性粒细胞［％，*M*（范围）］	5（0～19）	4.5（0～19）	5（0～19）	6（0～16）	3.980	0.137
脾脏肋缘下［cm，*M*（范围）］	1（0～32）	0（0～32）	4.5（0～25）	4（0～23）	21.087	<0.001
合并症［例（％）］	627（32.9）	527（34.2）	60（23.4）	40（37.0）	12.359	0.002
ELTS危险度［例（％）］					16.385	0.012
低危	1 234（64.7）	1 018（66.0）	154（60.2）	62（57.4）		
中危	456（23.9）	358（23.2）	72（28.1）	26（24.1）		
高危	152（8.0）	111（7.2）	27（10.5）	14（13.0）		
不详	64（3.4）	55（3.6）	3（1.2）	6（5.6）		
随访时间［月，*M*（*IQR*）］	41（14～80）	48（17～84）	33（16～66）	8（4～13）	163.422	<0.001

二、严重血细胞减少发生情况

TKI治疗中，发生≥3级血细胞减少的患者共有186例（9.8％），其中白细胞减少41例（2.2％），血小板减少114例（6％），白细胞和血小板均减少31例（1.6％），中位WBC最低值1.57（0.09～1.97）×10^9^/L，中位PLT最低值26（1～49）×10^9^/L。中位发生时间为TKI治疗1（0.2～3）个月，中位持续时间0.6（0.1～10.6）个月。146例（7.7％）因≥3级血细胞减少停药，中位停药时间为0.6（0.1～5.6）个月，其中56例（38.4％）持续时间≤2周，以初始剂量恢复用药。90例（61.6％）停药时间>2周，以初始剂量的2/3～3/4恢复用药。其中32例（21.9％）在后续治疗中逐渐恢复原剂量，38例（26.0％）维持低剂量用药，20例（13.7％）因减量后仍反复出现血细胞减少而换药。

三组患者TKI相关的≥3级血细胞减少的发生率（*P*＝0.854）、发生时间（*P*＝0.690）以及持续时间（*P*＝0.200）差异均无统计学意义，但服用伊马替尼患者4级血液学毒性的发生率（3.0％）显著低于尼洛替尼（5.5％）和氟马替尼（7.4％）（与尼洛替尼组比较，*P*＝0.002；与氟马替尼组比较，*P*＝0.007），而发生时间（*P*＝0.11）以及持续时间（*P*＝0.712）差异无统计学意义（表2）。

**表2:** 三组初发成人慢性髓细胞性白血病患者严重血细胞减少发生情况比较

严重血细胞减少发生情况	总体 （1 906例）	伊马替尼 （1 542例）	尼洛替尼 （256例）	氟马替尼 （108例）	统计量 （*χ^2^*/*H*）	*P*值
≥3级						
发生率（％）	9.8	9.6	10.2	11.1	0.316	0.854
发生时间［月，*M*（范围）］	1.0（0.2～3.0）	1.0（0.2～3.0）	1.3（0.4～2.7）	1.0（0.5～2.8）	0.741	0.690
持续时间［月，*M*（范围）］	0.6（0.1～10.6）	0.6（0.1～10.6）	0.7（0.1～3.4）	1.6（0.1～5.6）	3.220	0.200
4级						
发生率（％）	3.6	3.0	5.5	7.4	13.732	0.001
发生时间［月，*M*（范围）］	1.2（0.2～3.0）	1.2（0.2～3.0）	1.0（0.5～2.7）	0.7（0.5～1.7）	4.345	0.114
持续时间［月，*M*（范围）］	0.8（0.1～5.8）	0.9（0.2～5.8）	0.7（0.1～3.3）	1.8（0.1～5.1）	0.678	0.712

三、PSM分析

在服用二代TKI的364例患者中，以年龄、性别、全血细胞计数、外周血原始细胞及嗜碱性粒细胞比例、ELTS评分和合并症作为匹配协变量，以2∶1比例PSM匹配后，共纳入241例患者，其中尼洛替尼组152例，氟马替尼组89例，两组患者基线特征差异无统计学意义（**扫描本文首页二维码查阅附表1**）。两组≥3级血细胞减少的发生率分别为13.8％和12.4％（*P*＝0.748），4级分别为9.3％和7.9％（*P*＝0.714），差异均无统计学意义。

将服用二代TKI的患者合并后与伊马替尼患者（1 542例）按上述匹配协变量以1∶3比例进行PSM匹配，最终纳入1 178例患者，其中伊马替尼组875例，二代TKI组303例，两组患者基线特征差异无统计学意义（**扫描本文首页二维码查阅附表2**）。两组≥3级血细胞减少的发生率分别为9.8％和10.9％（*P*＝0.597），而二代TKI组4级血细胞减少的发生率显著高于伊马替尼组（7.3％对2.7％，*P*<0.001）。

四、发生严重血细胞减少的相关因素

1. 伊马替尼：采用ROC曲线确定初诊时脾脏大小、WBC和HGB对于≥3级血细胞减少的最佳截断值，取整后分别为：脾脏肋缘下5 cm，WBC 129×10^9^/L和HGB 110 g/L。年龄、PLT、外周血原始细胞比例和外周血嗜碱性粒细胞比例因无法得出有意义的截断值，故采用中位数为截断值，分别为42岁、410×10^9^/L、1％和5％。

当纳入初诊时所有基线特征（不包括ELTS评分）时，多因素分析显示，女性、诊断时HGB≤110 g/L、脾脏肋缘下≥5 cm与发生≥3级血细胞减少显著相关（表3）。

**表3:** 初发成人慢性髓细胞性白血病患者伊马替尼相关严重血细胞减少多因素分析

因素	纳入所有基线因素		纳入ELTS和其他基线因素	
	*P*值	*OR*（95％*CI*）	*P*值	*OR*（95％*CI*）
女性	0.039	1.5（1.1～2.1）	0.069	1.4（1.0～2.0）
WBC≥129×10^9^/L			0.036	1.5（1.1～2.3）
HGB≤110 g/L	0.003	1.8（1.2～2.8）	0.003	1.9（1.2～2.8）
脾脏（肋缘下<5 cm为参照）	0.043			
≥5 cm	0.024	1.6（1.1～2.4）		
未知	0.742	0.9（0.4～1.8）		

根据上述变量回归系数各赋1分，将信息完整的1 396例患者分为低危组（0～1分，926例，66.3％）和高危组（2～3分，470例，33.7％）。两组≥3级血细胞减少的发生率分别为6.6％（95％*CI*：5.0％～8.2％）和16.0％（95％*CI*：12.6％～19.3％）（*P*<0.001），4级血细胞减少的发生率分别为1.3％（95％*CI*：0.6％～2.0％）和5.7％（95％*CI*：3.6％～7.9％）（*P*<0.001）。以低危组为参考，高危组发生≥3级血细胞减少的*OR*值为2.7（95％*CI*：1.9～3.9），两组≥3级和4级血细胞减少的发生时间和持续时间差异均无统计学意义。

当纳入ELTS时，其他基线变量包括性别、WBC、HGB、外周血嗜碱性粒细胞比例和合并症，多因素分析显示：女性、诊断时WBC≥129×10^9^/L和HGB≤110 g/L为发生≥3级血细胞减少的危险因素。将危险因素各赋1分后，将1 396例患者分为低危组（0～1分，881例，63.1％）和高危组（2～3分，515例，36.9％）。两组≥3级血细胞减少的发生率分别为6.7％（95％*CI*：5.0％～8.4％）和15.0％（95％*CI*：11.9％～18.0％）（*P*<0.001），4级血细胞减少的发生率分别为1.2％（95％*CI*：0.5％～2.0％）和5.4％（95％*CI*：3.5％～7.4％）（*P*<0.001）。以低危组为参考，高危组发生≥3级血细胞减少的*OR*值为2.4（95％*CI*：1.7～3.5），两组≥3级和4级血细胞减少的发生时间和持续时间差异均无统计学意义。

比较两种纳入不同变量（分别纳入所有基线特征或ELTS）的预测模型（图1），两种预测结果相似。

**图1:**
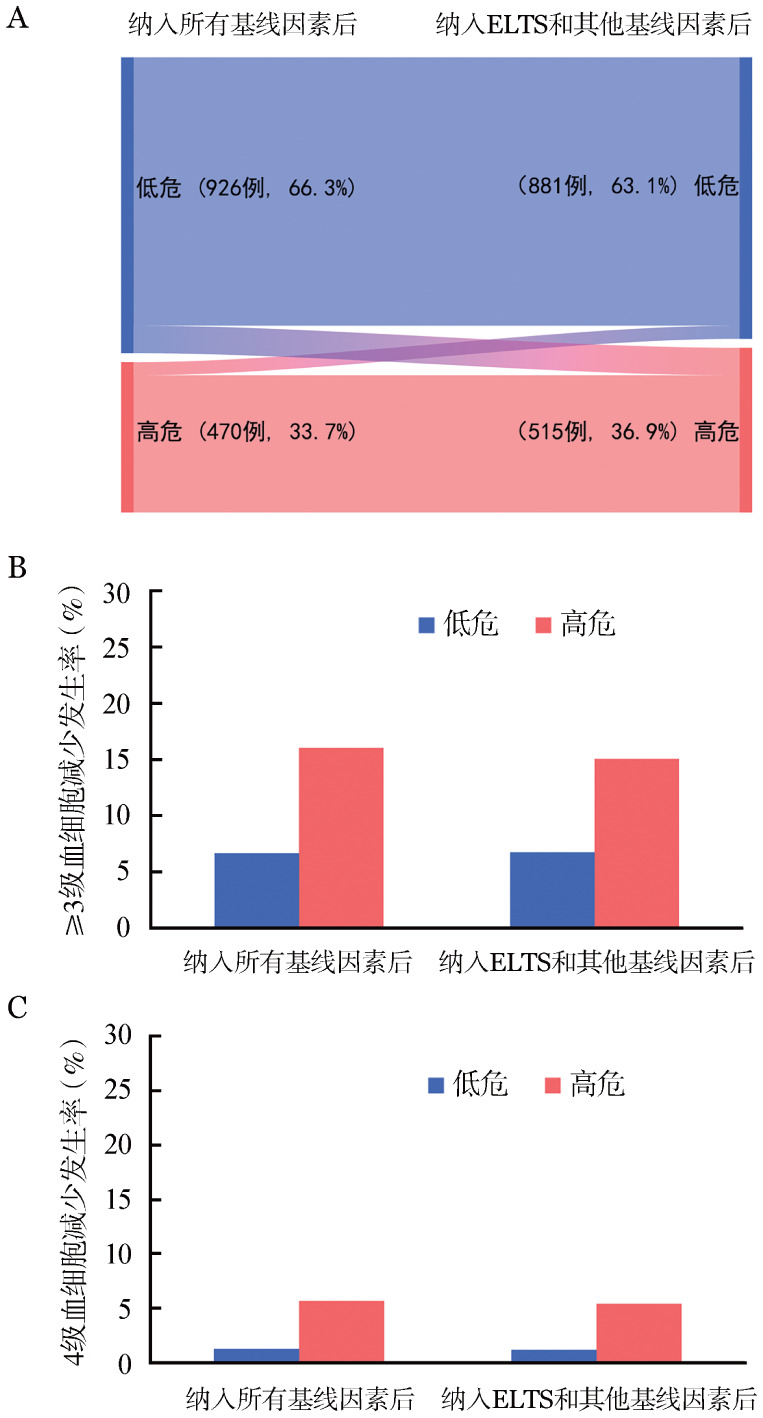
初发成人慢性髓细胞性白血病患者发生伊马替尼相关严重血细胞减少两种预测模型比较 **A** 桑基图；**B** ≥3级血细胞减少；**C** 4级血细胞减少

2. 二代TKI：采用ROC曲线确定初诊时脾脏肋缘下大小、WBC、HGB和外周血原始细胞对于≥3级血细胞减少的最佳截断值，取整后分别为：脾脏肋缘下5 cm，WBC 129×10^9^/L，HGB 100 g/L和外周血原始细胞2％。年龄、PLT和外周血嗜碱性粒细胞比例无法得出有意义的截断值，故采用中位数为截断值，分别为37岁、430×10^9^/L和5％。

当纳入初诊时所有基线特征（不包括ELTS评分），多因素分析显示，诊断时HGB≤100 g/L、脾脏肋缘下≥5 cm与发生≥3级血细胞减少显著相关（表4）。

**表4:** 初发成人慢性髓细胞性白血病患者二代酪氨酸激酶抑制剂相关严重血细胞减少的多因素分析

因素	纳入所有基线因素后		纳入ELTS和其他基线因素后	
	*P*值	*OR*（95％*CI*）	*P*值	*OR*（95％*CI*）
WBC≥129×10^9^/L			0.008	4.0（1.4～11.2）
HGB≤100 g/L	0.011	2.7（1.2～6.0）	0.003	3.3（1.5～7.1）
脾脏（肋缘下<5 cm为参照）	0.023			
≥5 cm	0.006	4.3（1.5～12.0）		
未知	0.455	1.8（0.4～8.0）		

根据上述变量回归系数赋分各赋1分，将信息完整的314例患者分组：低危组（0分，137例，43.6％），中危组（1分，104例，33.1％）和高危组（2分，73例，23.2％），三组≥3级血细胞减少的发生率分别为1.5％（95％*CI*：0～3.0％）、9.6％（95％*CI*：3.9％～15.4％）和30.1％（95％*CI*：19.4％～41.0％）（*P*<0.001），4级血细胞减少的发生率分别为0.7％（95％*CI*：0～1.5％）、4.8％（95％*CI*：0.6％～9.0％）和21.9％（95％*CI*：12.2％～31.6％）（*P*<0.001）。以低危组为参考，中、高危组发生≥3级血细胞减少的*OR*值分别为7.2（95％*CI*：1.5～33.5）和29.1（95％*CI*：6.6～128.2），两组≥3级和4级血细胞减少的发生时间和持续时间差异均无统计学意义。

当纳入ELTS时，其他基线变量同伊马替尼，多因素分析显示：诊断时WBC≥129×10^9^/L、HGB≤100 g/L为发生≥3级血细胞减少的危险因素。将危险因素各赋1分后，将314例患者分为低危组（0分，128例，40.8％），中危组（1分，101例，32.2％）和高危组（2分，85例，27.1％）。三组≥3级血细胞减少的发生率分别为2.3％（95％*CI*：0～4.7％）、6.9％（95％*CI*：1.9％～12.0％）和28.2％（95％*CI*：18.5％～38.0％）（*P*<0.001），4级血细胞减少的发生率分别为0.8％（95％*CI*：0～1.6％）、4.0％（95％*CI*：0.1％～7.8％）和20.0％（95％*CI*：11.3％～28.7％）（*P*<0.001）。

比较两种纳入不同变量（分别纳入所有基线特征或ELTS）的预测模型（图2），可见各组人群差异较小，两种预测模型的预测结果相似。

**图2:**
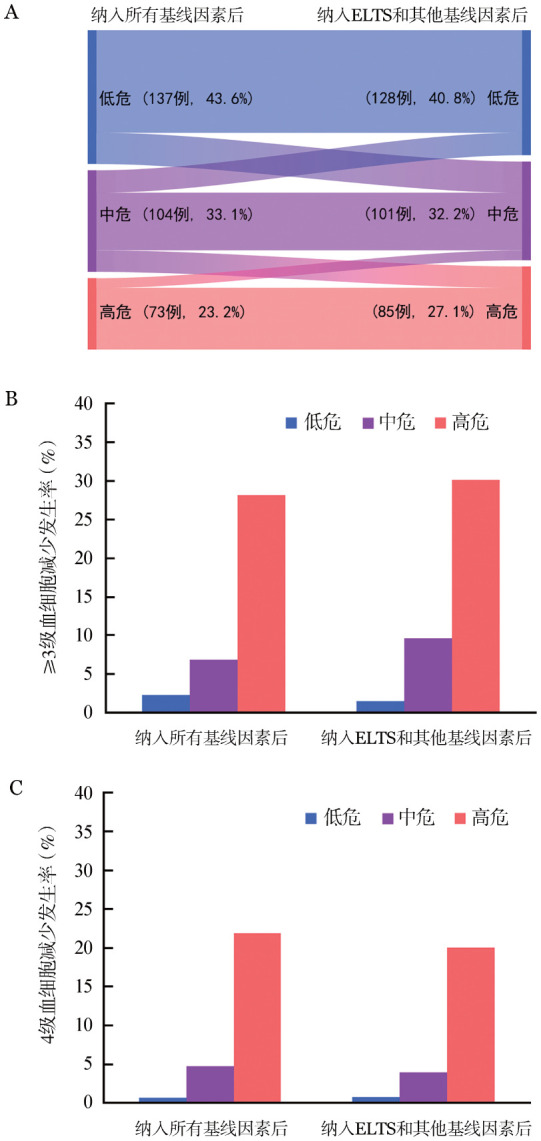
初发成人慢性髓细胞性白血病患者发生二代酪氨酸激酶抑制剂相关严重血细胞减少两种预测模型比较 **A** 桑基图；**B** ≥3级血细胞减少；**C** 4级血细胞减少

## 讨论

按照国内外指南推荐，当患者发生≥3级血液学不良反应需要减量、停用TKI，因此我们需要更严密地监测高危患者严重血细胞减少的发生。本研究显示，近10％的患者在一线伊马替尼、尼洛替尼或氟马替尼治疗早期发生严重血细胞减少，且以血小板减少为主，女性、贫血和脾大（或白细胞计数高）是服用TKI患者发生≥3级血细胞减少的危险因素。

多项研究报道，TKI治疗中≥3级白细胞减少和（或）血小板减少的发生率在伊马替尼400 mg/d时为9％～24％[10]–[12]、尼洛替尼300 mg每日两次时为9.8％～15％[10],[12]–[13]，氟马替尼600 mg/d时为3％～24％[14]–[16]，本研究结果与其相似，但以血小板减少为主。与ENESTnd、ENESTchina和FESTnd的研究结果一致，伊马替尼与二代TKI严重血细胞减少的发生率差异无统计学意义，但我们发现二代TKI相关的4级血细胞减少发生率显著增高。因此，对于使用二代TKI治疗的患者更需密切监测严重血液学不良反应的发生。

既往关于严重血液学不良反应影响因素的研究多以伊马替尼治疗的患者为对象，发现初诊时HGB<79 g/L、中性粒细胞绝对计数<1.5×10^9^/L、PLT<150×10^9^/L或>450×10^9^/L与血细胞减少的发生显著相关[17]–[18]。本中心既往研究显示，女性、诊断时WBC≥100×10^9^/L和CP-Sokal高危、原始细胞增多型加速期是伊马替尼相关严重血细胞减少的高危因素[5]，男性、初诊时贫血、年龄高和脾大与达沙替尼、尼洛替尼相关严重血细胞减少的发生显著相关[19]。本研究发现，除了女性、WBC高和贫血，脾大也与伊马替尼相关的严重血细胞减少相关；除了贫血和脾大，WBC高与尼洛替尼或氟马替尼一线治疗的严重血细胞减少相关。有研究报道，服用TKI患者中，女性血药浓度高于男性，这可能是女性易发严重血液学不良反应的原因之一[20]。TKI治疗过程中出现血液学不良反应主要有以下原因：①治疗过程中，尤其在治疗初期，异常的Ph ^+^白血病细胞克隆和正常造血均会受到抑制，正常的造血干/祖细胞需要时间恢复正常造血。②TKI在抑制BCR::ABL的同时，也会影响c-Kit基因、PDGFR基因及相关激酶，而这些基因和相关激酶在正常造血细胞生长发育和关键信号转导中起到重要作用[21]。二代TKI较伊马替尼对Ph ^+^白血病细胞克隆的抑制性更强，这可能导致正常造血受抑更加明显，增加4级血液学不良反应发生风险[13]–[14]。

本研究存在以下局限性：①回顾性研究；②服用二代TKI的患者相对较少；③在使用PSM对3种TKI相关严重血细胞减少的发生率分析后，尼洛替尼和氟马替尼组患者特征仍可能存在偏倚。

严重血细胞减少是TKI治疗的CML-CP患者常见的不良事件，二代TKI相较一代4级血液学不良反应更为显著。女性、血红蛋白水平低和脾大（或白细胞计数高）是TKI相关严重血细胞减少的影响因素。通过预测模型识别高危人群，针对此人群在治疗早期严密监测，并及时予以干预。

## Supplementary Material


