# Supplementary figures and images for: Variables associated with severe cytopenia in adult chronic phase chronic myeloid leukemia patients receiving initial tyrosine kinase inhibitors
Source: Zhonghua Xue Ye Xue Za Zhi. 2026 Mar;47(3):234–40. [Article in Chinese] doi: 10.3760/cma.j.cn121090-20251023-00478 (PMC13103592; doi:10.3760/cma.j.cn121090-20251023-00478)

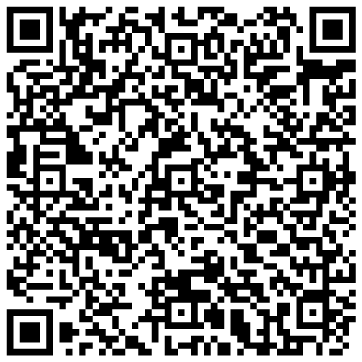

Supplement: Supplementary file 1 [file cjh-47-03-234-g003.tif]
